# γ-Hydroxybutyrate does not mediate glucose inhibition of glucagon secretion

**DOI:** 10.1074/jbc.RA119.009577

**Published:** 2020-03-10

**Authors:** Qian Yu, Bao Khanh Lai, Parvin Ahooghalandari, Anders Helander, Erik Gylfe, Patrick Gilon, Anders Tengholm

**Affiliations:** ‡ Department of Medical Cell Biology, Uppsala University, SE-751 23 Uppsala, Sweden; § Université Catholique de Louvain, Institute of Experimental and Clinical Research, Pole of Endocrinology, Diabetes and Nutrition, 1200 Brussels, Belgium; ¶ Department of Laboratory Medicine, Karolinska Institutet, and Clinical Pharmacology and Clinical Chemistry, Karolinska University Laboratory, SE-141 86 Stockholm, Sweden

**Keywords:** calcium, cyclic AMP (cAMP), glucagon, insulin secretion, pancreatic islet, alpha-cell, GHB, glucose homeostasis, somatostatin

## Abstract

Hypersecretion of glucagon from pancreatic α-cells strongly contributes to diabetic hyperglycemia. Moreover, failure of α-cells to increase glucagon secretion in response to falling blood glucose concentrations compromises the defense against hypoglycemia, a common complication in diabetes therapy. However, the mechanisms underlying glucose regulation of glucagon secretion are poorly understood and likely involve both α-cell–intrinsic and intraislet paracrine signaling. Among paracrine factors, glucose-stimulated release of the GABA metabolite γ-hydroxybutyric acid (GHB) from pancreatic β-cells might mediate glucose suppression of glucagon release via GHB receptors on α-cells. However, the direct effects of GHB on α-cell signaling and glucagon release have not been investigated. Here, we found that GHB (4–10 μm) lacked effects on the cytoplasmic concentrations of the secretion-regulating messengers Ca^2+^ and cAMP in mouse α-cells. Glucagon secretion from perifused mouse islets was also unaffected by GHB at both 1 and 7 mm glucose. The GHB receptor agonist 3-chloropropanoic acid and the antagonist NCS-382 had no effects on glucagon secretion and did not affect stimulation of secretion induced by a drop in glucose from 7 to 1 mm. Inhibition of endogenous GHB formation with the GABA transaminase inhibitor vigabatrin also failed to influence glucagon secretion at 1 mm glucose and did not prevent the suppressive effect of 7 mm glucose. In human islets, GHB tended to stimulate glucagon secretion at 1 mm glucose, an effect mimicked by 3-chloropropanoic acid. We conclude that GHB does not mediate the inhibitory effect of glucose on glucagon secretion.

## Introduction

Glucagon secreted from pancreatic α-cells is the principal glucose-elevating hormone counteracting hypoglycemia by stimulating glucose production in the liver. This critical counter-regulatory action is often impaired in diabetes (1). Moreover, diabetic patients frequently exhibit abnormally high postprandial glucagon release, contributing to hyperglycemia (2). However, the mechanisms by which glucose regulates glucagon secretion are poorly understood, and fundamentally different hypotheses have been proposed (reviewed in Ref. 3). It is known that the glucose concentration is monitored by neurons, which also regulate glucagon secretion from the α-cells (4, 5). Nevertheless, glucose inhibition of glucagon secretion from the perfused pancreas (6) and isolated islets (7, 8) indicates that glucagon release is also regulated without neural influences. Glucagon secretion may thus be controlled by paracrine factors from other cells within the islets or by α-cell–intrinsic glucose sensing (3, 9–12).

During hyperglycemia when β-cells are active, paracrine β-cell factors can be envisaged to contribute to inhibition of glucagon secretion. Because insulin has been found to inhibit glucagon release, insulin may seem a likely paracrine mediator of glucose-inhibited glucagon release (13). However, a paracrine role of insulin in the physiologically important glucagon counterregulation of hypoglycemia seems unlikely because insulin release is basal under such conditions. The neurotransmitter GABA (14–16) and its metabolite γ-hydroxybutyrate (GHB)^3^ (17), proposed to be released from glucose-stimulated β-cells, have also been implicated in paracrine control of glucagon secretion. GABA inhibits glucagon secretion by opening GABA_A_-receptor chloride channels (14–16). However, both the total content and release of GABA from β-cells are reduced by glucose elevation in rat islets (18), and in human islets, GABA is released from β-cells in a glucose-independent manner (19), making GABA an unlikely paracrine mediator of glucose-inhibited glucagon secretion.

GHB is a well-established neurotransmitter in the central nervous system (CNS) and has been in clinical use for the treatment of narcolepsy and alcohol dependence, but it has also been abused as a recreational drug (20–22). The pharmacology of GHB is complex, and there are both low- and high-affinity targets, but their molecular identities are not fully elucidated. The GABA_B_ receptor is a low-affinity target mediating the effects of pharmacological doses of GHB (23). GABA_A_ receptors and a specific GHB receptor have recently been proposed to mediate the effects of low concentrations of GHB (24–27). In pancreatic islets, GHB is formed in the GABA shunt pathway via GABA transaminase that generates succinate semialdehyde, which is converted to GHB by succinate semialdehyde reductase (17). The release of GHB from β-cells has been found to be stimulated already by glucose elevation from 0 to 5 mm (17). GHB may therefore be the factor that terminates the counterregulatory glucagon response when normoglycemia is reached. Indeed, glucagon secretion from human islets stimulated by glycine or an amino acid mixture was suppressed by the GHB agonist 3-chloropropionate, and glucose inhibition of amino acid-stimulated secretion was reversed by the antagonist NCS-382 (17). Additional arguments for the involvement of GHB in paracrine regulation of glucagon secretion came from studies of human islets from type 2 diabetic donors, which lost their glucose responsiveness in parallel with a loss of GABA shunt enzymes (17). The knowledge about GHB in islets is largely based on the use of synthetic agonists and antagonists of the GHB receptor, but the direct effects of GHB on α-cells and GHB-induced intracellular signaling have not been previously studied. In the present study, we therefore investigated the effects of GHB on the exocytosis-regulating messengers Ca^2+^ and cAMP in α-cells, as well as on glucagon secretion from mouse and human pancreatic islets.

## Results

### GHB has little effect on submembrane Ca^2+^ and cAMP concentrations in mouse α-cells

**Figure 1.**
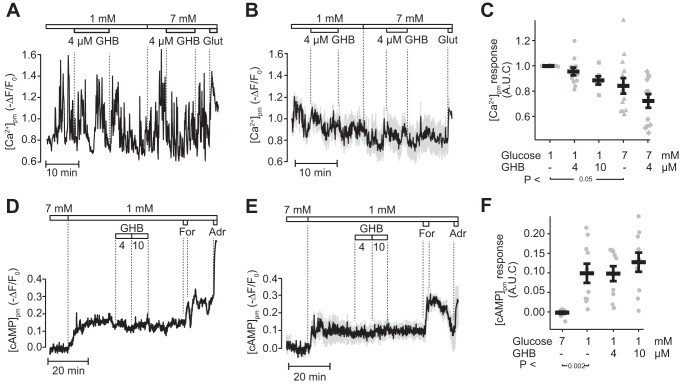
**GHB has little effect on [Ca^2+^]_pm_ and [cAMP]_pm_ in α-cells in intact mouse islets.** *A*, TIRF recording of [Ca^2+^]_pm_ from a mouse α-cell exposed to 4 μm GHB in the presence of 1 and 7 mm glucose. Glutamate (*Glut*; 1 mm) was added for cell identification. The data are representative for twelve cells from three independent islet isolations. *B*, average [Ca^2+^]_pm_ (*black*) ± S.E. (*gray*) from five α-cells within same islet treated as in *A. C*, individual data points along with averages ± S.E. for the time-average [Ca^2+^]_pm_ signal at different concentrations of glucose and GHB. *D*, effects of reduction of the glucose concentration from 7 to 1 mm and addition of 4 or 10 μm GHB, 10 μm forskolin (*For*), and 10 μm adrenaline (*Adr*) on [cAMP]_pm_ in a single α-cell within a mouse islet. The data are representative for nine cells from three recordings with islets from three independent isolations. *E*, average [cAMP]_pm_ data (*black*) ± S.E. (*gray*) from six α-cells within the same islet treated as in *D. F*, individual data points along with averages ± S.E. for the time-average [cAMP]_pm_ level in mouse α-cells (*n* = twelve from three independent experiments). The *p* values refer to statistical comparisons with Student's *t* test.

The concentrations of Ca^2+^ and cAMP in the sub–plasma membrane space ([Ca^2+^]_pm_ and [cAMP]_pm_) are critical for the regulation of glucagon secretion (12), and we therefore explored whether GHB affects [Ca^2+^]_pm_ and [cAMP]_pm_ in individual α-cells within intact mouse islets using fluorescent reporters and total internal reflection fluorescence (TIRF) microscopy. In the presence of 1 mm glucose, α-cells loaded with the Ca^2+^ indicator Fluo-4 showed vivid irregular [Ca^2+^]_pm_ spiking (Fig. 1*A*), consistent with stimulation of glucagon secretion under these conditions. α-Cell identity was verified by the prompt and sustained increase of [Ca^2+^]_pm_ induced by 1 mm glutamate (28). The addition of 4 or 10 μm GHB had no significant effect on [Ca^2+^]_pm_ in individual α-cells (Fig. 1*A*), on the averaged signal from several α-cells within the same islet (Fig. 1*B*), or on the time-averaged [Ca^2+^]_pm_ from all α-cell recordings (Fig. 1*C*). As previously reported (28), increase of the glucose concentration to 7 mm induced a transient interruption of [Ca^2+^]_pm_ signaling in some cells, and the time-average level was consequently slightly decreased (Fig. 1*C*). GHB lacked significant effect also in the presence of 7 mm glucose (Fig. 1, *A–C*). Because the GHB receptor has been reported to induce a G-protein–mediated lowering of cAMP (25, 29), we next explored whether GHB affected [cAMP]_pm_ in the α-cells. As previously reported (12), lowering the glucose concentration from 7 to 1 mm significantly increased [cAMP]_pm_ in α-cells identified by adrenaline-induced elevation [cAMP]_pm_. However, there was no effect of 4 or 10 μm GHB in α-cells subsequently responding to 10 μm forskolin and 10 μm adrenaline with pronounced [cAMP]_pm_ elevations (Fig. 1, *D–F*).

### GHB does not affect glucagon secretion from mouse islets

**Figure 2.**
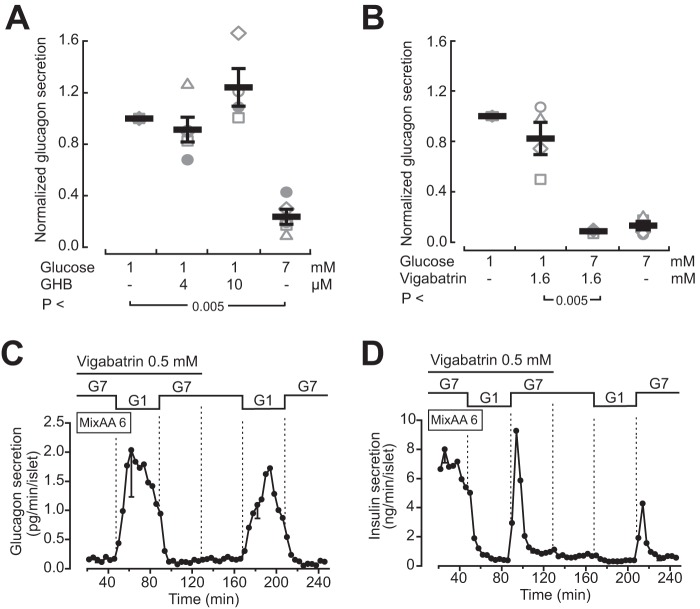
**GHB does not mediate glucose inhibition of glucagon secretion.** *A*, glucagon secretion from perfused mouse islets sequentially exposed to different concentrations of glucose and GHB. Data from five independent experiments are presented along with averages ± S.E. *B*, as in *A*, but with the addition of vigabatrin (1.6 mm). The data and averages ± S.E. from four independent experiments are shown. The *p* values refer to statistic comparison with Student's *t* test. *C* and *D*, glucagon (*C*) and insulin (*D*) secretion measured in parallel from perfused mouse islets exposed to a 6 mm amino acid mixture and different concentrations of glucose. The islets were pretreated overnight with 2 mm vigabatrin, and 0.5 mm of the compound was present during the beginning of the perfusion as indicated. *Traces* are the means ± S.E. for three experiments with islets from different preparations.

To investigate the direct effect of GHB on glucagon secretion, we used a slow perfusion setup to measure glucagon release from batches of 10–15 mouse islets sequentially exposed to different conditions. GHB at 4 μm had no consistent effect on glucagon secretion at 1 mm glucose (Fig. 2*A*). At 10 μm, GHB tended to stimulate glucagon release slightly, but this effect was not statistically significant. In contrast, when the glucose concentration was increased from 1 to 7 mm, glucagon secretion was inhibited by ∼80% (*p* < 0.005; Fig. 2*A*).

### Inhibition of endogenous GHB production does not alter glucagon secretion

We also attempted to interfere with endogenous GHB production from the GABA shunt in islet cells by inhibiting GABA transaminase with vigabatrin. Acute exposure to 1.6 mm vigabatrin, a concentration at least 10 times above its IC_50_ for GABA transaminase (30), tended to weakly reduce glucagon secretion at 1 mm glucose but did not prevent the glucagon inhibition by 7 mm glucose (Fig. 2*B*). To potentially facilitate detection of the modulatory effects of pharmacological agents, we employed an alternative protocol to assess glucagon secretion in which batches of 200 islets were continuously exposed to buffer containing a 6 mm amino acid mixture to increase glucagon secretion. Overnight culture with 2 mm vigabatrin did not prevent the ability of glucose to modulate glucagon release. Accordingly, when the glucose concentration was lowered from 7 to 1 mm, there was a pronounced increase of glucagon secretion, which was reversed when the concentration was restored to 7 mm (Fig. 2*C*). Insulin secretion measured from the same islets showed the opposite response with lowering during glucose reduction and transient stimulation of secretion upon introduction of 7 mm of the sugar (Fig. 2*D*).

### An agonist and an antagonist of the GHB receptor lack effect on glucagon and insulin secretion from mouse islets

**Figure 3.**
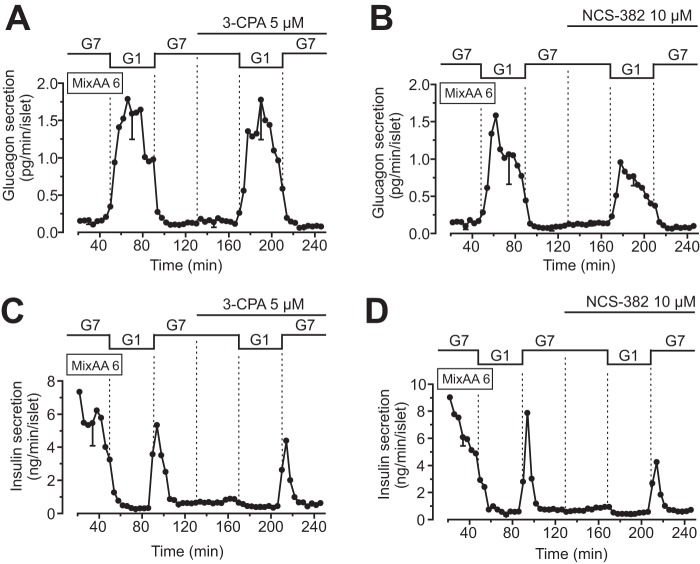
**Effects on glucagon and insulin secretion by a GHB receptor agonist and antagonist.** *A* and *B*, glucagon secretion from perfused mouse islets exposed to an amino acid mixture and sequential changes of the glucose concentrations in the absence or presence of the GHB receptor agonist 3-CPA (5 μm) and the antagonist NCS-382 (10 μm). As the latter compound was dissolved in DMSO, the vehicle was added to the control buffer to maintain a constant concentration of 0.1% throughout the experiment. *Traces* are the means ± S.E. for three experiments with islets from different preparations. *C* and *D*, insulin secretion from the same islets as in *A* and *B*. The values are means ± S.E. for three experiments with islets from different preparations.

To further evaluate the impact of GHB receptor signaling on glucagon secretion, we used the GHB receptor agonist 3-chloropropanoic acid (3-CPA). In the presence of 6 mm amino acid mixture, glucagon secretion increased when reducing the glucose concentration from 7 to 1 mm. However, 3-CPA (5 μm) neither affected glucagon secretion at 7 mm glucose nor prevented the stimulation by 1 mm of the sugar (Fig. 3*A*). Similarly, the GHB receptor antagonist NCS-382 (10 μm) had little effect on the glucose-regulated glucagon secretion (Fig. 3*B*). Measurements of insulin release from the same samples showed transient stimulation when glucose was increased from 1 to 7 mm with no obvious effects of GHB receptor activation or inhibition (Fig. 3, *C* and *D*). Together, these data indicate that paracrine GHB signaling does not modulate glucagon secretion in mouse islets.

### GHB and its receptor agonist increases glucagon secretion in human islets

**Figure 4.**
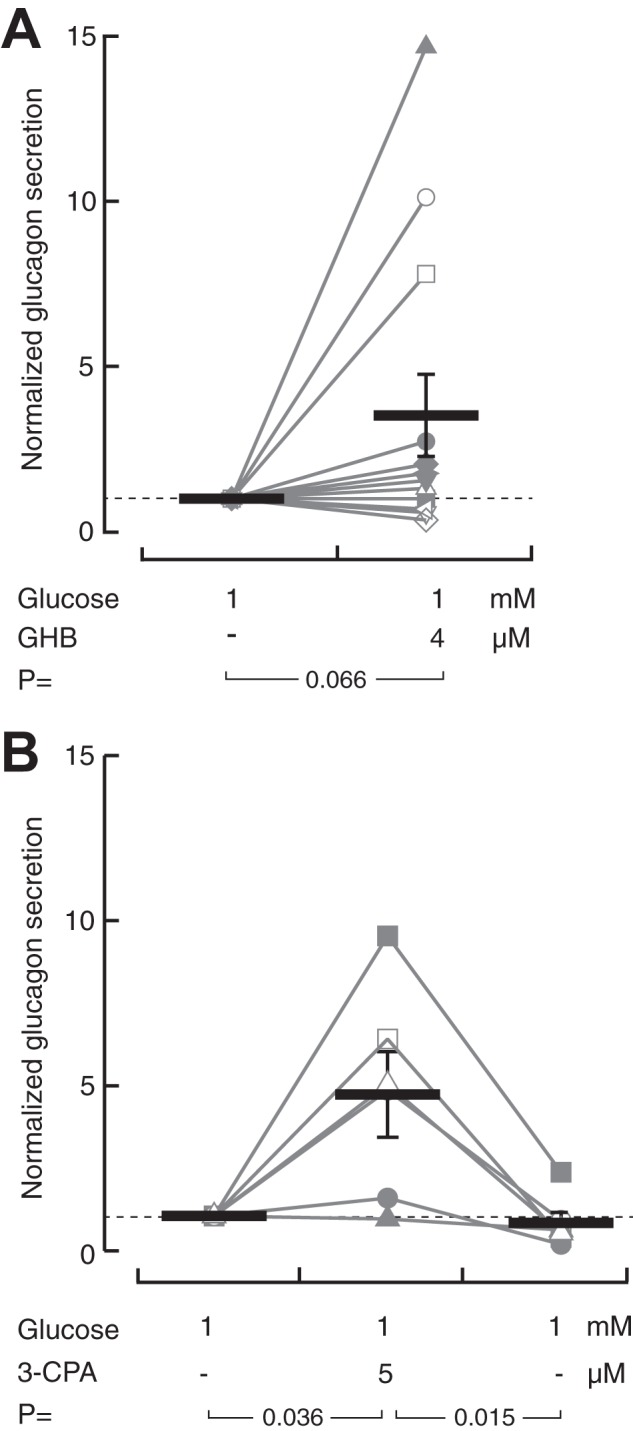
**GHB promotes somatostatin-dependent glucagon secretion from human islets.** *A*, normalized glucagon secretion from human islets exposed to 1 mm glucose with or without 4 μm GHB. Shown is a scatter plot with means ± S.E. for twelve experiments with islets from twelve different preparations. The *p* value refers to statistical comparison with Student's paired *t* test. *B*, as in *A* but with 5 μm of the GHB agonist 3-CPA. Shown is a scatter plot with means ± S.E. for six experiments with islets from six different preparations.

The direct effects of GHB were investigated also in human islets. In the presence of 1 mm glucose, GHB (4 μm) showed a variable response. In 3 of 12 preparations, glucagon release was slightly suppressed. In islets from one donor, GHB had no effect, and in the remaining 8 preparations, there was at least 30% stimulation of glucagon secretion. The average from all experiments was 3.7 ± 1.3-fold stimulation (*p* = 0.066; *n* = 12; Fig. 4*A*). Activation of GHB receptors with 5 μm 3-CPA showed a more consistent stimulatory effect (4.7 ± 1.3; *n* = 6; *p* = 0.036), which was reversed following washout of the drug (Fig. 4*B*).

## Discussion

The mechanisms underlying glucose regulation of glucagon secretion remain controversial. Both α-cell–intrinsic and paracrine mechanisms may contribute and their relative importance depend on the prevailing glucose concentration (3). The present study challenges the previously suggested role of the neurotransmitter GHB released from β-cells and acting on α-cells to mediate glucose-inhibition of glucagon secretion (17). We demonstrate that such inhibition by GHB neither occurs in mouse nor in human islets and that the transmitter rather has a stimulatory effect on glucagon secretion from human islets.

GHB is a well-known inhibitory neurotransmitter in the CNS (20, 21). GHB is also generated in pancreatic β-cells and glucose elevation has been found to stimulate production and release of GHB that parallels inhibition of glucagon release from human islets (17). However, the direct effects of GHB have previously not been studied, possibly because its use as an illicit recreational drug (22) has hampered GHB availability. Moreover, the nature of putative GHB receptor(s) in islets has not been clarified. In the CNS, there are both high-affinity (nano- to micromolar) and low-affinity (millimolar) binding sites for the transmitter (21). We chose concentrations of GHB expected to activate its high-affinity receptors but not GABA receptors. If GHB is a paracrine inhibitor of glucagon secretion, one would expect fast effects on exocytosis-regulating steps. Because Ca^2+^ controls exocytosis in α-cells (31), we investigated GHB effects on [Ca^2+^]_pm_. At low-glucose concentrations that stimulate glucagon secretion, α-cells exhibited fast, irregular Ca^2+^ oscillations, but this pattern was unaffected by GHB. A slight reduction of the average [Ca^2+^]_pm_ level (Fig. 1*C*) was likely due to a time-dependent decline in the signal caused by photobleaching and leakage of the Ca^2+^ indicator from the cells. However, the lack of effect on Ca^2+^ signaling does not exclude that GHB inhibits secretion by other mechanisms. cAMP is another messenger that acts by enhancing Ca^2+^-dependent exocytosis. In α-cells, this amplifying action of cAMP may perhaps be even more important than changes in Ca^2+^, and recent studies have indeed highlighted cAMP as the main mediator of glucose-regulated glucagon secretion (12, 32). However, GHB did not lower [cAMP]_pm_, which instead tended to increase at the highest concentration tested.

In accordance with the lack of effects on Ca^2+^ and cAMP signaling, GHB failed to influence glucagon release from mouse islets. Two different approaches were used to measure secretion, one of which employed slow perifusion of a small number of islets to catch principal effects without dynamic details. This method proved robust and allowed detection of glucose-induced changes in glucagon secretion. We also used a protocol with a larger number of islets exposed to an amino acid mixture to increase secretion and catch its dynamic aspects. Irrespective of the approach, we did not find significant effects of GHB addition, the specific GHB receptor agonist 3-CPA, the antagonist NCS-382, or interference with GHB production with the GABA transaminase inhibitor vigabatrin. The concentrations of the drugs were the same as those reported to be effective in a previous study (17), and in the case of vigabatrin, we exposed islets both acutely and treated them overnight with the drug to compensate for low membrane permeability.

At variance with the lack of effect in mouse islets, GHB and its receptor agonist tended to stimulate glucagon secretion from human islets, but there were marked differences between different islet preparations. These observations nevertheless contradict the conclusion from previous studies that GHB is a negative regulator of glucagon secretion (17). The discrepancy between the studies may reflect different experimental conditions. In the present study, the islets were perifused and not exposed to glycine or other amino acids. The experiments were designed to test whether GHB can mimic the suppression of glucagon secretion by glucose, which was clearly not the case for most islet preparations. It is an open question whether the stimulation observed in many preparations reflects a physiologically significant effect, and the underlying mechanism is unknown. Because GHB generally is an inhibitory neurotransmitter (20, 21), it is tempting to speculate that its effect on α-cells is indirect and mediated for example via suppression of somatostatin release, which has a tonic, inhibitory effect on glucagon secretion (8).

The reason for the different GHB responses in mouse and human islets may be species differences in expression of the GHB receptor involved, which is difficult to verify without knowledge of its molecular identity. Mouse and human islets differ in cell composition and architecture with human islets showing a mosaic of the different cell types throughout the islet, whereas mouse islets have a β-cell–rich islet core surrounded by a mantle of α- and δ-cells (33). The human islet architecture may therefore be more favorable for paracrine signaling between β-cells and non–β-cells. It is also possible that the extent of paracrine signaling varies with the size of the mouse islets, because the fraction of δ-cells is higher in small islets, and that of α-cells is higher in large islets (34).

In conclusion, the present data change the view that GHB released from β-cells contributes to the inhibitory effect of glucose on glucagon secretion. If anything, GHB tends to stimulate glucagon release, at least in human islets.

## Experimental procedures

### Materials

Penicillin, streptomycin, glutamine, fetal calf serum, and the acetoxymethyl ester of the Ca^2+^ indicator Fluo-4 were from Thermo Fisher Scientific. HEPES, poly-l-lysine, 3-CPA and glutamate were from Sigma–Aldrich. Vigabatrin was from both Sigma–Aldrich and Tocris Bioscience (Biotechne Ltd., Abingdon, UK), NCS-382 was from Tocris Bioscience, and GHB was obtained from LGC GmbH (Luckenwalde, Germany). The experimental buffer used in islet imaging experiments contained 138 mm NaCl, 4.8 mm KCl, 1.2 mm MgCl_2_, 1.3 mm CaCl_2_, 3 mm glucose, 0.5 mg/ml albumin, and 25 mm HEPES with pH adjusted to 7.40 with NaOH.

### Islet isolation and culture

All animal handling and experimental procedures were approved by the Uppsala regional ethics committee and the Ethics Committee for Animal Experimentation of the Health Sciences Sector of the Université Catholique de Louvain (Project 2014/UCL/MD/016). Islets were isolated from 5 to 11 months old C57Bl/6J mice (Taconic, Denmark or Janvier Labs, France) and then cultured for 1–2 days in RPMI 1640 medium supplemented with 5.5 mm glucose (7 mm for dynamic secretion experiments), 10% fetal bovine serum, 100 μg/ml penicillin, and 100 μg/ml streptomycin at 37 °C in an atmosphere of 5% CO_2_ in humidified air.

Human islets from 14 normoglycemic cadaveric organ donors (Table 1) were obtained via the Nordic Network for Clinical Islet Transplantation in Uppsala. All experiments with human islets were approved by the Uppsala human ethics committee. The isolated islets were cultured for up to 7 days in CMRL 1066 culture medium containing 5.5 mm glucose, 100 units/ml penicillin, 100 μg/ml streptomycin, 2 mm glutamine, and 10% fetal bovine serum at 37 °C in an atmosphere of 5% CO_2_.

**Table 1:** **Human islet donor characteristics** Age, sex, body mass index (BMI) and glycated hemoglobin (HbA1c) of the 14 human pancreas donors from which islets were isolated.

Islet preparation	Age	Sex	BMI	HbA1c
	*Years*		*kg/m^2^*	*mmol/mol (%)*
1	38	Male	25.8	32 (5.1)
2	26	Female	34.9	35 (5.4)
3	57	Male	21	38 (5.6)
4	73	Male	28.1	34 (5.3)
5	73	Male	26	34 (5.3)
6	64	Female	29.4	33 (5.2)
7	55	Male	30.5	46 (6.4)
8	64	Female	33.6	39 (5.7)
9	65	Male	30.1	42 (6.0)
10	71	Male	22.4	41 (5.9)
11	81	Female	21.1	34 (5.3)
12	74	Female	22.7	34 (5.3)
13	39	Male	26.6	34 (5.3)
14	70	Female	40.8	42 (6.0)

### Recordings of [Ca^2+^]_pm_ and [cAMP]_pm_

For [Ca^2+^]_pm_ recordings, islets were preincubated for 30–40 min in experimental buffer containing 138 mm NaCl, 4.8 mm KCl, 1.2 mm MgCl_2_, 1.3 mm CaCl_2_, 3 mm glucose, 0.5 mg/ml albumin, and 25 mm HEPES (pH 7.40) and supplemented with 1.6 μm of the acetoxymethyl ester of the Ca^2+^ indicator Fluo-4. After washing, the islets were attached to a poly-l-lysine–coated coverslip in an open 50-μl chamber and superfused with experimental buffer at a rate of 0.2 ml/min at 37 °C. A cAMP translocation reporter (35) comprised of a plasma membrane–targeted PKA regulatory RIIβ subunit and a PKA catalytic Cα subunit fused to mCherry was used for [cAMP]_pm_ measurements. The islets were incubated in culture medium with cAMP reporter adenoviruses at a concentration of 1–2 × 10^6^ plaque-forming unit/islet for 1–2 h. After the infection, the islets were washed with RPMI 1640 medium and cultured in complete medium for at least 18 h. Before imaging, the islets were preincubated for 30 min in experimental buffer.

Changes of [Ca^2+^]_pm_ and [cAMP]_pm_ were monitored with TIRF microscopy using a Nikon Ti microscope equipped with a 60×, 1.49-NA objective. Fluo4 and mCherry were excited with 488- and 561-nm lasers, respectively (Cobolt, Stockholm, Sweden) and led by an optical fiber into the TIRF illuminator attached to the microscope. Emission light was selected using the following filters (center wavelength/half-bandwidth): 527/27 nm for Fluo4 (Semrock, Rochester, NY) and 620-nm long-pass for mCherry (Melles Griot, Didam, The Netherlands) and detected with an Orca-ER digital CCD camera (Hamamatsu, Japan). Image analysis was performed with MetaFluor software (Molecular Devices Corp, Sunnyvale, CA). The [Ca^2+^]_pm_ signals were presented as fluorescence intensity (*F*) normalized to the average of the fluorescence at the initial condition (*F*_0_). The cAMP reporter fluorescence signal was inverted to obtain a positive relationship with [cAMP]_pm_ and expressed as −Δ*F*/*F*_0_, where Δ*F* = *F* − *F*_0_. α-Cells were identified based on their small footprint, [Ca^2+^]_pm_ signaling pattern at 1 mm glucose, [Ca^2+^]_pm_ response to glutamate (28), and [cAMP]_pm_ response to adrenaline (36).

### Measurements of glucagon and insulin secretion

In Figs. 2 (*A* and *B*) and 4, glucagon secretion was measured from batches of 10–15 islets, which were placed in a 10-μl chamber consisting of a Teflon tube (inner diameter, 1.07 mm; Habia Teknofluor, Knivsta, Sweden) with a Spectrum^TM^ Spectra Mesh^TM^ woven filter (polyether ether ketone, 35-μm mesh opening; Fisher Scientific) to prevent islets from escaping. The chamber was sealed with custom-made end plugs through which the perifusion tubing was attached. The chamber was perifused with experimental buffer (as for microscopy experiments but with 2.6 mm CaCl_2_) supplemented with different stimuli at a rate of 60 μl/min using a pressurized air system (AutoMate Scientific, Berkeley, CA). Before sample collection, the islets were perifused in buffer with 3 mm glucose for 30 min. The perfusate was subsequently collected every 5 min with 3–5 samples per experimental condition. Glucagon was measured with an ELISA kit (Mercodia, Uppsala, Sweden), and the results are presented as the averages of the samples at each condition, excluding the first sample, which might be influenced by the previous experimental condition.

The experiments in Figs. 2 (*C* and *D*) and 3 were performed with batches of ∼200 islets, perifused at a flow rate of 0.5 ml/min, with a buffer containing 124 mm NaCl, 4.8 mm KCl, 2.5 mm CaCl_2_, 1.2 mm MgCl_2_, 20 mm NaHCO_3_, 1 mg/ml BSA, 2 mm alanine, 2 mm glutamine, and 2 mm arginine (denoted “MixAA 6”) and test agents as indicated. It was gassed with O_2_:CO_2_ (95:5%; pH 7.4). Radioimmunoassays were used for parallel measurements of glucagon (Millipore) and insulin (custom designed).

### Statistics

The data are presented as means ± S.E. Statistical comparisons were performed with Student's *t* test.

## Author contributions

Q. Y., B. K. L., P. A., E. G., P. G., and A. T. formal analysis; Q. Y., B. K. L., P. A., and P. G. investigation; Q. Y. and A. T. writing-original draft; Q. Y., A. H., E. G., P. G., and A. T. writing-review and editing; A. H., P. G., and A. T. resources; E. G., P. G., and A. T. conceptualization; P. G. and A. T. funding acquisition; P. G. and A. T. project administration; A. T. supervision.

## Footnotes

3 The abbreviations used are:

GHB: γ-hydroxybutyric acid
CNS: central nervous system
[Ca^2+^]_pm_: Ca^2+^ in the sub–plasma membrane space
[cAMP]_pm_: cAMP in the sub–plasma membrane space
3-CPA: 3-chloropropanoic acid
TIRF: total internal reflection fluorescence.
